# Measles outbreak in a unique sociocultural context: epidemiological investigation and control measures in Northern Brazil, 2025

**DOI:** 10.1590/S1678-9946202668065

**Published:** 2026-10-02

**Authors:** Rebeca Porto Rosa, Rita de Cássia Ferreira Lins, Marcelo Yoshito Wada, Tiara Bruna Teixeira Teodosio, Ana Catarina de Melo Araújo, Amanda Campos Feitosa, Cintia Paula Vieira Carrero, Cinthya Resplandes, Corinto Gomes dos Santos, Danielle Menezes de Brito Mota, Elena de Carvalho Cremm Prendergast, Eliana Ribeiro de Oliveira, Gisele Silva Carvalho Luz, Josafá do Nascimento Cavalcante, Lucimeire Neris Sevilha da Silva Campos, Pedro Pereira da Silva, Marli Jerônimo da Silva Rodrigues, Greice Madeleine Ikeda Carmo, Eder Gatti Fernandes, Marielly Reis Resende Sousa, Marilda M. Siqueira, Xenia Rawena Lemos, Suelen Soares da Silva, Elaine da Silva Castro Ferreira, Bruno Silva Milagres, Karen Machado Gomes

**Affiliations:** 1 Ministério da Saúde, Secretaria de Vigilância em Saúde e Ambiente, Departamento do Programa Nacional de Imunizações, Brasília, Distrito Federal, Brazil; 2 Secretaria de Saúde do Estado do Tocantins, Palmas, Tocantins, Brazil; 3 Secretaria Municipal de Saúde de Campos Lindos, Campos Lindos, Tocantins, Brazil; 4 Ministério da Saúde, Secretaria de Vigilância em Saúde e Ambiente, Coordenação-Geral de Laboratórios de Saúde Pública, Brasília, Distrito Federal, Brazil; 5 Fundação Oswaldo Cruz, Laboratório de Vírus Respiratórios, Exantemáticos, Enterovírus e Emergências Virais, Rio de Janeiro, Rio de Janeiro, Brazil

**Keywords:** Disease outbreaks, Measles, Vaccine hesitancy, Public health surveillance.

## Abstract

After the recertification of measles elimination in Brazil, outbreaks associated with imported cases may occur in pockets of susceptible individuals, including communities with low vaccination coverage. This study describes an outbreak investigation conducted in 2025 in Campos Lindos, Tocantins State, Brazil. In total, 25 cases were confirmed, mostly among unvaccinated Old Believers (a traditional community of Russian origin) following viral introduction linked to international travel, with transmission occurring predominantly within the community. Outbreak response measures, including ring vaccination and active case finding, interrupted transmission after 12 weeks with no new cases. The rapid response was effective and reinforces the importance of identifying susceptibility pockets in the population to prevent measles reintroduction.

## INTRODUCTION

Brazil lost its measles elimination status in 2019 following virus reintroduction and sustained transmission for more than 12 months. The country was then recertified in November 2024 after strengthening surveillance and immunization activities¹. In this context, outbreaks have occurred in several countries that had previously achieved measles-free status, including in North and South America, highlighting the risk of reintroduction associated with international mobility and vaccine hesitancy^2^ ^,^ ^3^. In July 2025, a measles outbreak occurred in the municipality of Campos Lindos, Tocantins State, after the return of a family from Bolivia, a country with active measles transmission^4^. Although the municipality had a predominantly vaccinated population, cases were concentrated in a specific group.

## MATERIALS AND METHODS

A descriptive study was conducted among residents of Campos Lindos, a located in Tocantins State, northern Brazil (13°02’S, 46°46’W), with an economy predominantly based on agriculture and an estimated population of 8,653 inhabitants in 2025^5^. This study describes the outbreak, including the affected population, immunization aspects, surveillance activities, laboratory investigation, and prevention and control measures. Analysis ranged from July 18 to September 9, 2025, with retrospective case findings from June 18 onward and surveillance extending up to December 15 due to the international 12-week period required after the last reported case.

This epidemiological investigation included interviews with suspected and confirmed cases, clinical sample collection, contact tracing and monitoring, active community case finding, review of medical records from the two Brazilian Unified Health System health services in the municipality, immunization activities, and training of health professionals. This investigation aims to identify the disease, the source of infection, and its transmission chain and to implement measures to control the outbreak and prevent community transmission.

To control the outbreak and prevent new cases, immunization activities using measles-containing vaccines (MMR, MMRV, or MR) were carried out in individuals aged six months to 59 years according to the National Immunization Schedule. The strategies included (a) ring vaccination within 72 h after contact with suspected or confirmed cases, (b) vaccination sweep around confirmed cases (covering a minimum radius of five blocks in urban areas or five properties in rural areas), and (c) intensification of vaccination across the entire municipal population.

To analyze vaccination in the context of the outbreak, vaccine doses administered from July to December 2025 in all age groups were considered according to records from the National Health Data Network^6^. Municipal vaccination coverage was evaluated in children aged 1 to <2 years following guidance from the Brazilian Ministry of Health, using the number of vaccinated children in this age group × 100 as the numerator and the corresponding estimated population as the denominator.

Data were obtained from the Notifiable Diseases Information System^7^, the Laboratory Environment Manager, the Citizen Electronic Health Record, and the National Health Data Network vaccination dashboard. Descriptive analyses were performed using Microsoft Office, and the transmission chain was constructed on Go.Data^8^. Case definitions from the Brazilian Health Surveillance Guide were adopted^9^, and control actions followed Brazilian Ministry of Health protocols for outbreak response^10^ ^,^ ^11^.

Ethical aspects followed the provisions of Law Nº 8,080/1990 (which regulates epidemiological surveillance actions in Brazil) and Resolution Nº 510 of April 7, 2016, of the National Health Council as this was an outbreak investigation conducted exclusively in response to an imminent public health risk, according to Brazilian regulations for public health surveillance activities. The confidentiality of those involved in the investigation was ensured (no individual identification).

## RESULTS

From June 24 to 26, 2025, a family of four, residing in Campos Lindos, Tocantins, returned to Brazil by road after spending approximately one month in Bolivia (4). The primary case (the father, case 1) developed a rash on July 2 and was initially suspected of dengue fever at a local healthcare facility. His wife and two children (cases 2, 3, and 4) developed symptoms 10 days after the onset of the rash in case 1. They sought no medical care. The index case was identified from case 5, the family’s niece, who developed a rash on July 15 and sought medical care two days later. The measles case was then reported to the municipal surveillance service and, the following day, to the Tocantins State Health Department and the Ministry of Health. Given the risk of measles reintroduction in Brazil, the ongoing outbreak in Bolivia, and the travel history of the measles cases, a rapid and coordinated response was initiated in the three levels of governance (municipal, state, and federal) to investigate the local outbreak and implement control measures.

The epidemiological investigation found additional suspected cases among contacts of the primary case and their relatives, indicating potential transmission (as they were susceptible). Case 1 was laboratory-confirmed for measles by serology and polymerase chain reaction on July 18 and classified as imported. However, it was impossible to determine whether infections in cases 2, 3, and 4 were acquired during their stay in Bolivia or by contact with case 1 after returning to Brazil; therefore, they were classified as import-related cases.

In total, 26 cases were reported, of which one was discarded and 25 were confirmed over the 70-day surveillance period, with the highest incidence occurring in July 2025 (Figure 1). Among confirmed cases, 14 occurred in male patients (56%). The median age was 15 years, ranging from 1 to 30 years. Moreover, two cases required hospitalization. Both recovered.

Of the confirmed cases, 13 (52%) were confirmed by detectable polymerase chain reaction, two (8%) by reactive IgM serology, and 10 (40%) by epidemiological linkage due to the impossibility of sample collection. Samples with detectable polymerase chain reaction were sequenced, identifying genotype D8, lineage MVs/Ontario.CAN/47.24 (DSId 9171), consistent with documented viral circulation during the same period in Bolivia, Mexico, the United States, and Canada, confirming case importation.

**Figure 1:**
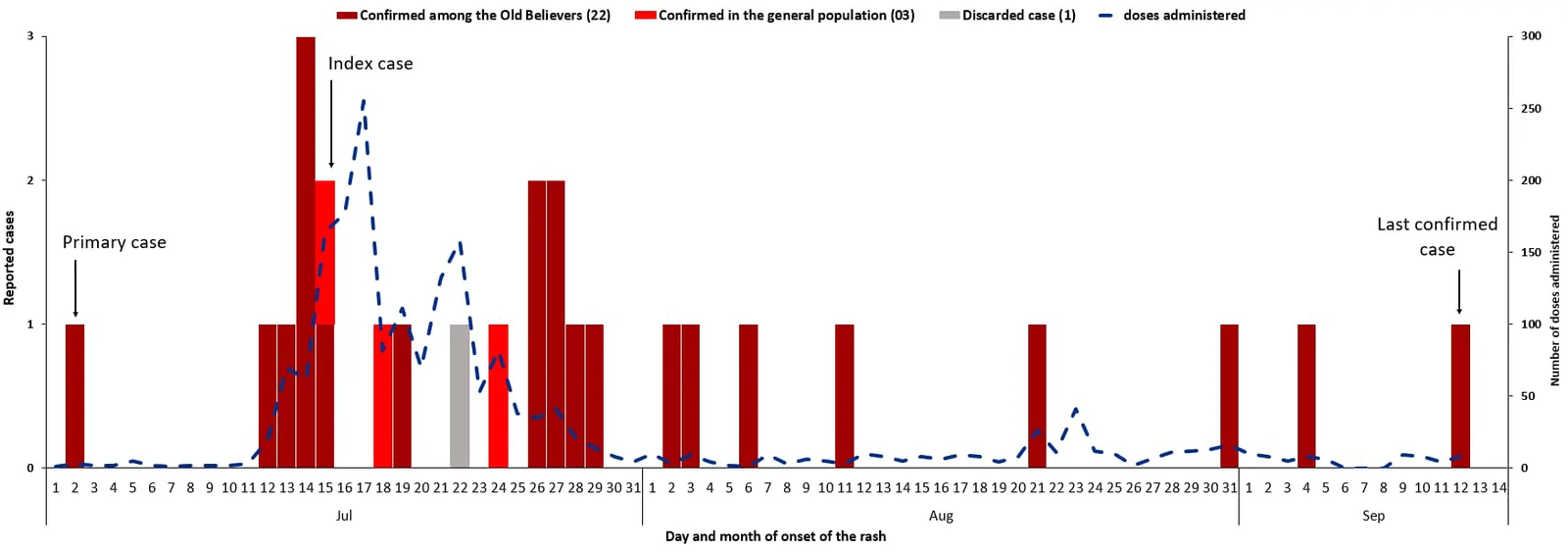
Confirmed measles cases and administered doses of measles-containing vaccines according to date of rash onset, Campos Lindos, Tocantins State, Brazil, 2025^6^ ^,^ ^7^(data obtained on December 23, 2025).

The transmission chain analysis showed that 84% (22) of the cases occurred in members of the traditional Russian-origin community known as the “Old Believers” (referred locally as “Russians”), of whom 91% had no record of measles vaccination. In total, three cases occurred in the general population of the municipality, including a health professional who provided care to the primary case and had no vaccination history. The other two cases, an infant and an adult, were also unvaccinated, and in both instances the source of infection could not be determined.

Considering the suspected cases and their initially asymptomatic contacts, 189 individuals were traced and monitored (Figure 2)^12^, mostly from the local commerce sector and health services, via home visits or telephone follow-up. The monitoring period spanned 30 days after the last exposure to a confirmed case for asymptomatic contacts or until the onset of signs and symptoms compatible with measles - at which point they were then considered suspected cases.

The investigation indicated that the outbreak began after the return of the primary case from Bolivia, with subsequent transmission to family members and a health professional, who generated new transmission chains. This study found the transmission chain for 22 (91.6%) secondary cases. However, it was impossible to identify any possible contact with confirmed cases for two cases from the general population.

Before the outbreak, the municipality had vaccination coverage of 123.33% for the first dose (D1) and 76.67% for the second dose (D2) in children aged under two years. Intensified vaccination activities targeting individuals aged six months to 59 years began after notification of the index case (case 5), including ring vaccination and vaccination sweep activities. Members of the Old Believers community received 17 doses, 27.9% of the estimated population (61 individuals) residing in urban and rural areas of the municipality.

Given the successive identification of cases, vaccine hesitancy within the Old Believers community, low D2 vaccination coverage, and the risk of sustained transmission in Brazil, a selective vaccination intensification strategy was implemented throughout the municipality to create a protective “cocoon” for individuals adhering to vaccination. A vaccination campaign day (“D-Day”) was organized using the two municipal primary healthcare facilities as fixed vaccination sites and a church and local food market as outreach sites since these locations had greater population circulation.

These strategies lead to the administration of 2,113 doses of measles-containing vaccines to individuals aged from six months up to 59 years in Campos Lindos, corresponding to 24.4% of its population. Among children under two years of age, vaccination coverage increased to 127.10% for D1 and 107.48% for D2, a 38% increase when compared with the pre-outbreak period.

To increase sensitivity in identifying unreported cases, a search for active community cases was conducted from July 20 to 30, 2025, including a retrospective investigation of signs and symptoms compatible with measles from July 1 onward. In total, six teams (24 professionals) visited 280 households and interviewed 410 individuals, finding no suspected cases. Active institutional case finding was also conducted in the health services of the municipality, manually and electronically reviewing 1,488 medical records, including the Citizen Electronic Health Record, without further cases being identified.

**Figure 2:**
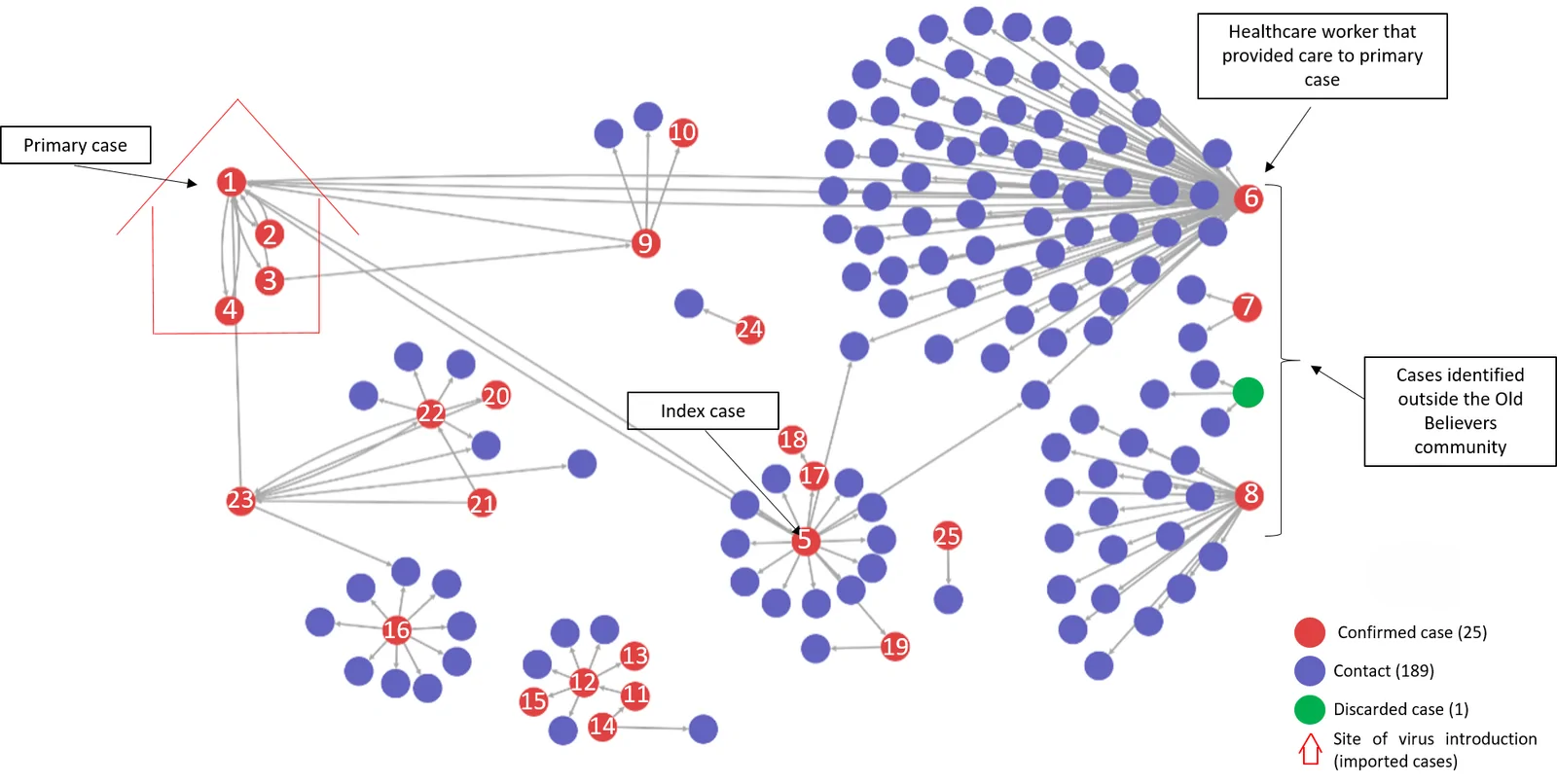
Transmission network of measles cases obtained from epidemiological investigation and contact tracing (Go.Data). Campos Lindos, Tocantins State, Brazil, 2025^8^ ^,^ ^12^(data up to November 4, 2025, subject to change).

In addition to the actions carried out in Campos Lindos, 603 health professionals from 49 municipalities in Tocantins State received training sessions on measles surveillance and outbreak response. Meetings were also held with health managers from four municipalities in Tocantins to strengthen awareness regarding surveillance and vaccination intensification in the region. Vaccination activities were intensified in surrounding municipalities, culminating in a statewide vaccination day across Tocantins state on August 9, 2025.

## DISCUSSION

The measles outbreak in Campos Lindos, Tocantins, involved 25 cases, concentrated among unvaccinated individuals from a traditional Russian-origin community (Old Believers) following viral reintroduction associated with international travel. Timely outbreak investigation, laboratory confirmation, and immunization strategies interrupted viral transmission.

The rapid increase in vaccination coverage was decisive in interrupting transmission and preventing the expansion of the outbreak to other municipalities. Vaccination coverage exceeding 100% likely reflects an underestimation of the population denominator stemming from the registration of live births in other municipalities, a common situation in locations without maternity healthcare services, such as Campos Lindos.

The adopted vaccination intensification strategies, based on intensive vaccination of populations surrounding identified cases and susceptible individuals, established a protective immunological buffer. Conceptually, this approach corresponds to “ring vaccination,” which aims to create zones of immunity around confirmed cases, interrupting further spread beyond the initial focus of transmission^13^. These strategies may be applied preventively (before epidemics occur) and in containment during outbreaks aiming to limit disease propagation^13^ ^,^ ^14^.

The outbreak was declared over after 12 weeks without new cases, on December 5, 2025, contributing to the maintenance of measles elimination in Brazil. The control of this outbreak stemmed from integrated strategies between the three levels of Brazilian Unified Health System governance and implemented actions in other municipalities in the Tocantins State and in a neighboring state.

## CONCLUSIONS

Old Believers communities maintain distinct religious practices, strong internal cohesion, rural and domestic lifestyles, and lower daily circulation in collective or public environments^15^. These communities occur in municipalities in Mato Grosso, Parana, and Goias states and in countries such as Bolivia, the United States, and Canada. Intense people interaction occurs between these communities, including via marriages, which increases the group’s transnational mobility. The investigated outbreak showed a high proportion of unvaccinated individuals and resistance to vaccination. Mobility between countries with active viral circulation, low vaccination coverage, and strong interaction within members of the community contributed to the increase in case numbers. Similar situations have been described in outbreaks among Amish communities and other groups with low vaccination adherence in North America^16^ ^,^ ^17^.

The outbreak showed the high transmissibility of the virus in susceptible populations and the risk of viral reintroduction in Brazil by international travelers. The retrospective identification of the primary case highlights the need for sensitive surveillance systems capable of early detection, immediate response, and appropriate differential diagnoses. Ring vaccination proved to be an effective strategy in contexts of vaccine hesitancy to contain transmission. Municipalities should, therefore, identify pockets of susceptible individuals, vaccinate them, and, when necessary, adopt this approach to contain further disease spread.

## Data Availability

The anonymized dataset generated during this study is available from the corresponding author upon reasonable request.
